# Micro-Shaping of Nanopatterned Surfaces by Electron Beam Irradiation

**DOI:** 10.3390/mi7040066

**Published:** 2016-04-13

**Authors:** Carlos Angulo Barrios, Víctor Canalejas-Tejero

**Affiliations:** 1 Instituto de Sistemas Optoelectrónicos y Microtecnología (ISOM), ETSI Telecomunicación, Universidad Politécnica de Madrid, Ciudad Universitaria s/n, 28040 Madrid, Spain; victorcanalejas@isom.upm.es; 2 Department of Photonics and Bioengineering, ETSI Telecomunicación, Universidad Politécnica de Madrid, Ciudad Universitaria s/n, 28040 Madrid, Spain

**Keywords:** micro/nanopatterning, electron-beam lithography, polycarbonate, compact disk, micro-optics, microfluidics, lab-on-CD

## Abstract

We show that planar nanopatterned thin films on standard polycarbonate (PC) compact discs (CD) can be micro-shaped in a non-contact manner via direct e-beam exposure. The shape of the film can be controlled by proper selection of the e-beam parameters. As an example of application, we demonstrate a two-dimensional (2D) array of micro-lenses/reservoirs conformally covered by an Al 2D nanohole array (NHA) film on a PC CD substrate. It is also shown that such a curvilinear Al NHA layer can be easily transferred onto a flexible polymeric support. The presented technique provides a new tool for creating lab-on-CD architectures and developing multifunctional (flexible) non-planar nanostructured films and surfaces.

## 1. Introduction

Compact disc (CD) technology has emerged as a powerful and cost-effective solution for sensing and diagnosis uses [1,2]. The combination of microfluidics and optical structures for sensing and analytical measurements on standard polycarbonate (PC) CDs has led to the demonstration of lab-on-CD configurations [3,4,5,6] that take advantage of commercially available CD-driver operation. CD rotation allows both, fluidic functions to be achieved via centrifugal pumping and optical scanning of the sensing elements on the CD surface.

The fabrication of high-performance miniaturized optical sensors on PC CDs usually requires the deposition of thin films, which can be carried out by conventional methods such as evaporation [7] and sputtering [8], and micro/nanopatterning of those films. For the latter, hot embossing and other imprinting techniques are widely used because of their simplicity, low cost and reproducibility, which makes them highly suitable for mass production [9,10]. However, if shaping (e.g., curving) of micro/nanopatterned thin films on PC CDs is desired—for example, for adding new optical and fluidic functionalities and/or improving device performance—the imprinting processes may not be appropriate. This is because of the unavoidable contact between mold and polymer, which can contaminate or even destroy the patterned thin film due to the application of excessive pressure and/or undesired adhesion problems. 

**Figure 1:**
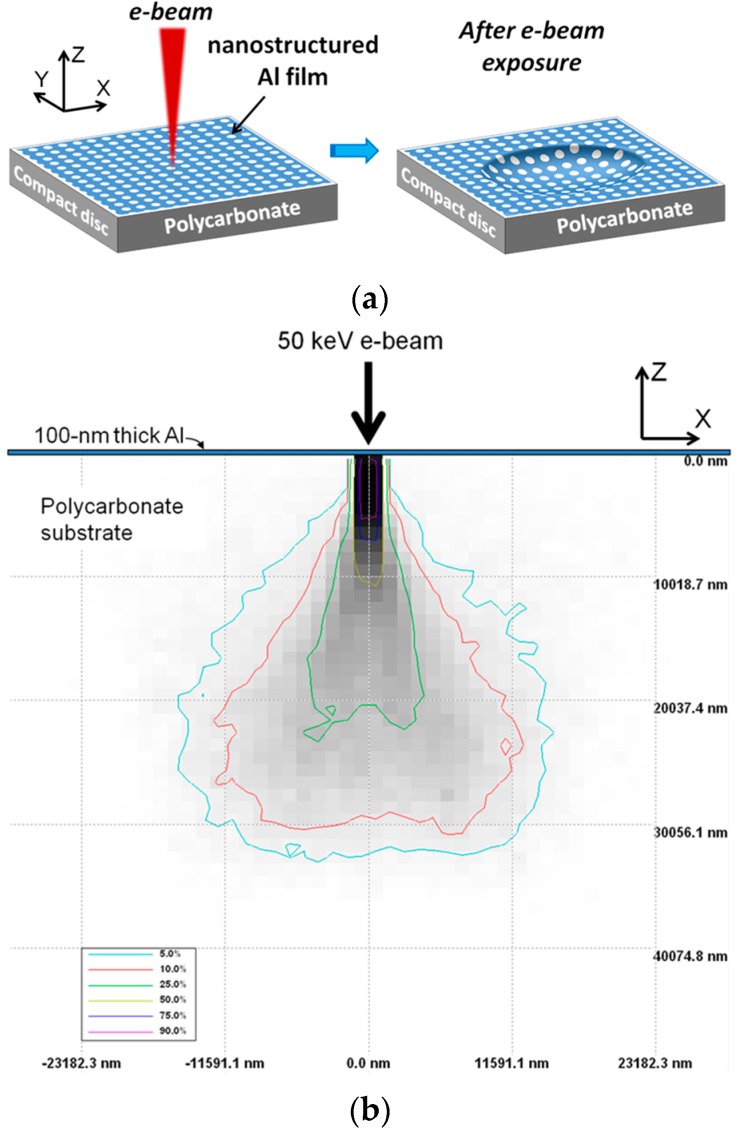
(**a**) Schematic diagram of the process for transforming a planar Al nanohole array (NHA) into a curvilinear geometry. Left: the Al NHA film, deposited on a polycarbonate (PC) compact disc (CD) substrate, is irradiated with an e-beam. Electrons cross the Al film and penetrate into the PC substrate, where their energy is used to crosslink the polymer. Right: cross-linking shrinks the exposed PC volume, curving the Al NHA inward. (**b**) Cross-sectional view of simulated absorbed energy in the PC substrate for a 50 keV 10-nm-radius e-beam. The gray shade ranges from light to dark as the absorbed energy density increases.

Here we introduce a non-contact approach to transform planar nanopatterned thin films on PC CDs into curvilinear geometries. In particular, we demonstrate that nanohole arrays (NHAs) drilled in Al thin films on PC CD substrates can be shaped by direct e-beam irradiation of the metal film surface. The method is schematically illustrated in Figure 1. It is based on the penetration of incident electrons into the PC substrate where their energy is absorbed. This leads to crosslinking of the irradiated polymer region [11] with consequent PC volume shrinking and film/surface deformation. The film curvature dependence on the main parameters of the shaping procedure is studied. As an example of application, we have fabricated a two-dimensional (2D) array of micro-lenses/reservoirs integrating a 2D NHA drilled in an Al film. Finally, we also demonstrate that the resulting non-planar nanopatterned metal surfaces on PC CD substrates can be easily transferred onto a flexible sticky tape by a simple *stick-and-peel* process developed recently [12].

## 2. Materials and Methods

### 2.1. Aluminum Nanohole Array Fabrication on PC CD Substrates

PC square substrates (12 mm × 12 mm) were cut from a 1.2-mm-thick standard CD (MPO Iberica, Madrid, Spain) using a dicing saw machine. The PC substrates were washed with detergent in ultrasonic bath, rinsed in deionized water (DIW) and isopropyl alcohol (IPA) and dried with N_2_ flow. Then, an e-beam-evaporated 100-nm-thick layer of Al was deposited on the flat surface (*i.e.*, with no track) of the PC CD substrates. Next, ZEP-520 positive-tone e-beam lithography (EBL) resist was spin-coated on the Al films at 5000 rpm. The samples were then immediately baked for 10 min at 120 °C. Dot matrix arrays of pitches 500 nm and 635 nm were patterned in the resist film by using a Crestec CABL-9000 C high-resolution EBL system (acceleration voltage = 50 kV, beam current = 1 nA). The exposed resist was developed at 0 °C for 40 s and N_2_ dried. Inductively coupled plasma (ICP) chemical dry etching was used to drill holes in the Al layer down to the PC substrate using the patterned ZEP-520 film as a mask. The ICP process was achieved using BCl_3_ (20 sccm) and Cl_2_ (10 sccm) gases and RF and ICP powers of 100 W. Immediately after the ICP etch, the samples were rinsed in DIW for 5 min to dissolve residual chloride ions. Finally, O_2_ plasma treatment was carried out to remove resist residues and passivate the Al surface [7].

### 2.2. Creation of Non-Planar Nanopatterned Al Surfaces on PC CD Substrates

The PC CD substrates containing the nanopatterned Al films were then subjected to direct e-beam irradiation of the metal films using the aforementioned EBL equipment (Crestec Corp., Tokyo, Japan) as shown schematically in Figure 1a. Circles of diameters (*d*_inc_) 2, 5 and 10 μm were written (scanned) with EBL at doses ranging from 12.8 to 320 mC/cm^2^.

### 2.3. Transfer Printing onto a Flexible Support

The PC CD substrates containing non-planar nanopatterned Al films were fixed on a glass slide by gluing the back side of the PC substrate to the glass with cyanocrylate-based glue (Loctite, Henkel Corp., Westlake, OH, USA). A general purpose transparent PSA tape (#550 Scotch^®^, 3M, St. Paul, MN, USA) was used for pattern transfer. A 19-mm-wide by approximately 4-cm-long piece of tape was applied onto the nanopatterned Al surface on PC using finger pressure for approximately 1 min. Then the adhesive tape was peeled off manually to transfer the non-planar metal film from the PC substrate onto the tape.

### 2.4. Optical Transmission Spectra Measurements

The transmission spectra of the Al NHAs on PC were measured at room temperature with a Jasco V-650 UV–VIS spectrophotometer (Jasco Analytical Instruments, Easton, MD, USA) with nonpolarized light under normal incidence in the 500–700 nm wavelength range and a spectral resolution of 0.2 nm.

### 2.5. Surface Characterization

Surface morphology was characterized by a FEI Inspect F50 scanning electron microscope (SEM) (FEI, Hillsboro, OR, USA), an Alpha-Step IQ mechanical profiler (KLA-Tencor Corp., Milpitas, CA, USA) and an Ambios Xi-100 3D interferometric optical profiler (LOT-QuantumDesign Ltd., Surrey, UK).

## 3. Results and Discussion

Figure 1b displays the cross-sectional distribution of the absorbed energy in the PC CD substrate calculated with a Monte Carlo simulation software (Université de Sherbrooke, Sherbrooke, QC, Canada) [13] for a 50 keV 10-nm-radius incident e-beam. The graph reveals that the electrons that cross the Al film and penetrate into the PC substrate are highly collimated up to a depth penetration of ~6 μm (dark region). As the electrons penetrate further they spread out, diminishing the absorbed energy density (light grey regions). PC crosslinking due to electron energy absorption should mostly take place in the dark area shown in Figure 1b. The resulting volume shrinkage of the crosslinked region ought to produce an inward bending of the PC surface—and, therefore, of the uplying Al film—around the directly irradiated region.

**Figure 2:**
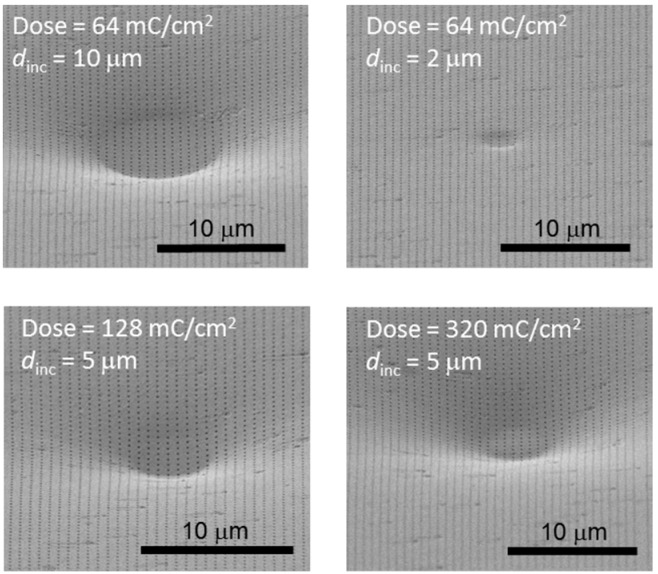
SEM photographs of the surface of an Al NHA film on a PC CD substrate, on which circles of different diameters (*d*_inc_) have been written directly by a 50 keV e-beam at several doses. Circular depressions of different widths and depths are created in the PC substrate, producing concave curvilinear Al NHA geometries.

This is corroborated in Figure 2, which shows SEM images of a 635-nm-period Al NHA film on a PC CD substrate on which circles with different *d*_inc_ values have been written directly by e-beam at several doses. It is seen that, after e-beam exposure, the patterned Al film is curved inwards, the directly irradiated circle being in the center of the circular depression. SEM analysis also revealed a uniform (un-cracked) curved Al NHA film for the studied EBL conditions.

**Figure 3:**
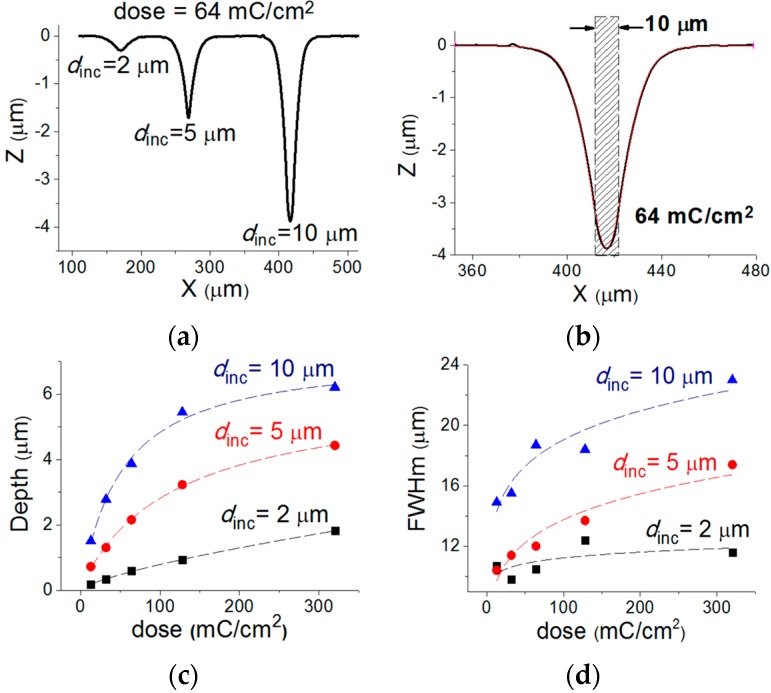
(**a**) Profiles along the diameter of three dips in an Al NHA/PC sample produced by e-beam irradiation at the same dose (64 mC/cm^2^) and different irradiated diameters (*d*_inc_): 2, 5 and 10 μm. (**b**) Profile of a single dip (black line) and double sigmoidal fit curve (red line). Dip depth (**c**) and FWHm (**d**)* vs.* electron dose for the considered irradiated diameters. Dashed lines correspond to sigmoidal fits of the measured data.

The profile along the diameter of the three dips created in the Al NHA/PC sample by e-beam irradiation at a dose of 64 mC/cm^2^ and different irradiated diameters (*d*_inc_ = 2, 5 and 10 μm) is shown in Figure 3a. Figure 3b depicts the profile for *d*_inc_ = 10 μm as a representative case. The profile shape can be well fitted to a symmetric double sigmoidal function (red line in Figure 3b: adjusted *R*^2^ = 0.999). Figure 3c,d plot the measured depth and full width at half minimum (FWHm) of the dips as a function of the electron dose for the three considered *d*_inc_ values. Both, depth and FWHm increase with both, the dose and *d*_inc_. This is expected since an increment of dose and/or *d*_inc_ boosts the amount of penetrated electrons and, therefore, the degree of PC crosslinking and surface deformation. The measurements in Figure 3c,d fit characteristic sigmoidal dose functions that saturate at large values of the dose. Saturation occurs because the number of PC molecules capable of absorbing the energy of the penetrated electrons is limited and due to the volume shrinking limitations of the polymeric material. The calibration sigmoidal curves of Figure 3c,d can be used to engineer the geometry and dimensions of a targeted curvilinear Al film by proper choice of the pattern layout (written circle diameter) and the electron dosage.

**Figure 4:**
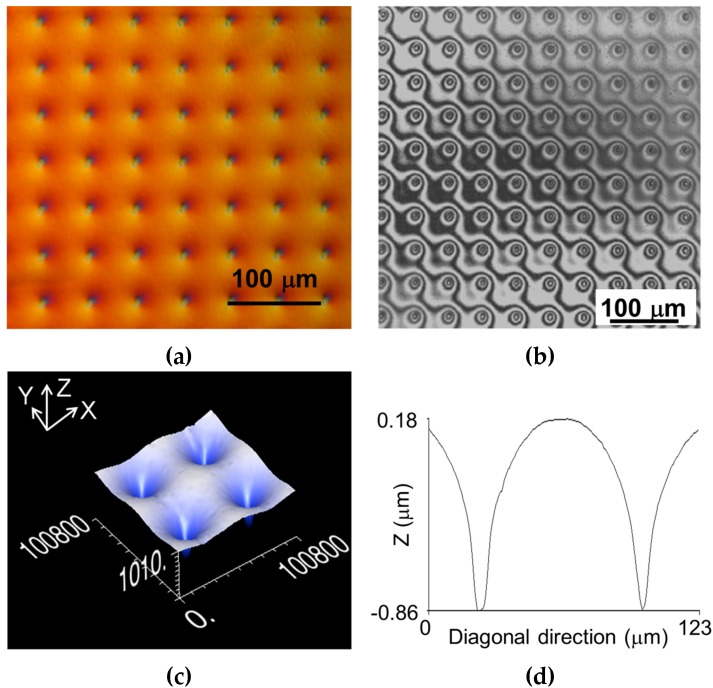
(**a**) Optical microscope photograph of a 2D square array of circular micro-dips (diverging microlenses and converging micromirrors) created by electron irradiation on a 500-nm-period Al NHA on PC CD. (**b**) Optical interference image of the surface; the micro-dips show typical circular interference patterns. (**c**) 3D optical profiler image of four neighboring micro-dips (scale is in nm) and (**d**) surface profile (cross-section of Figure 4c) along a diagonal of the dip square lattice unit.

Figure 4a shows an optical microscope photograph of a 50-μm-period 2D array of circular dips created by e-beam irradiation (*d*_inc_ = 5 μm, dose = 32 mC/cm^2^) of a 500-nm-period Al NHA on PC CD. Figure 4b–d correspond to surface characterization by interferometric optical profiler. The dips exhibit clear concentric interference patterns (Figure 4b) that are typical of curvilinear (concave and convex) circular surfaces. Note that the microdips can operate as both, diverging microlenses in transmission and converging micromirrors in reflection. The depth and width (FWHm) of the microlenses (Figure 4c,d) were 1.04 and 11 μm, respectively. Note also that the surface among the dips is curved, but outwards, as a consequence of the neighboring dip proximity. This illustrates a method to create convex nanostructured film geometries using the presented e-beam irradiation procedure.

**Figure 5:**
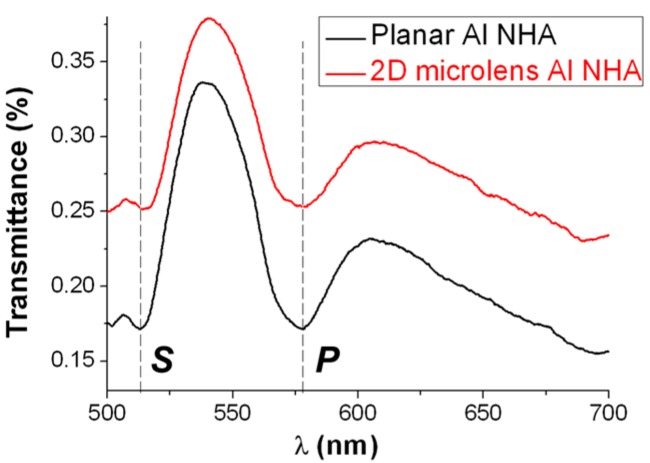
Measured transmittance spectra of a 500-nm-pitch Al NHA on PC-CD before (black line) and after (red line) the fabrication of a 2D array of concave microlenses by electron irradiation. *S* and *P* minima are associated to the excitation of SPPs at the metal-air and metal-substrate interfaces, respectively.

Figure 5 plots the spectral transmittance of the 500-nm-period Al NHA on PC-CD substrate before and after the creation of the microlenses. Both spectra exhibit similar spectral features. Minima denoted as *S* and *P* are attributed to the excitation of surface plasmon polaritons (SPPs) at the air-metal and substrate-metal interfaces, respectively [7]. Thus, the 2D microlens array with the integrated NHA preserves the plasmonic effects due to the nanostructured Al film besides adding optical characteristics of diverging lenses. Note also that the dips can operate as micro-reservoirs for optical interrogation of small volumes of liquid samples, which makes this configuration suitable for implementing CD-based microfluidic microarrays for multiplex analysis of biomolecules.

**Figure 6:**
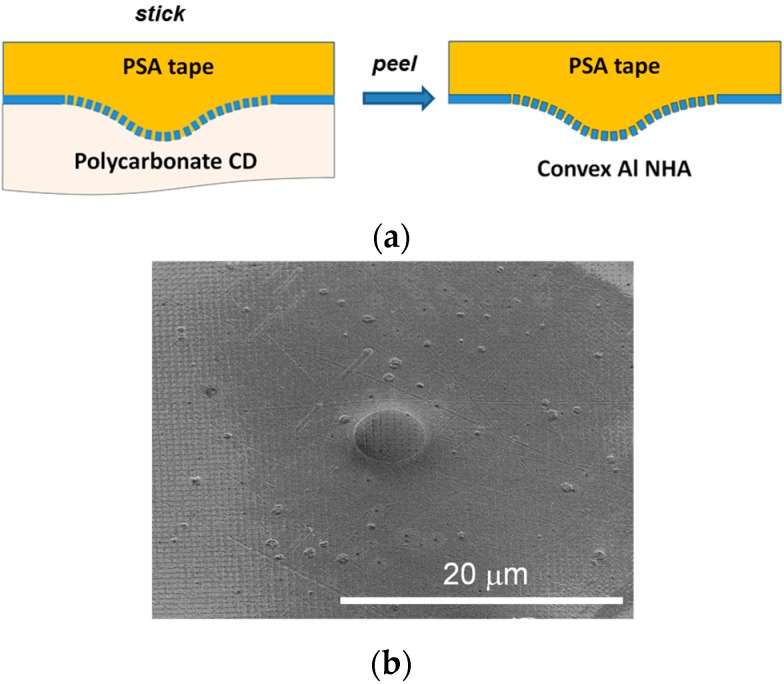
(**a**) Schematic diagram of the transfer of a concave Al NHA film from a PC CD substrate onto a pressure-sensitive adhesive (PSA) tape by a simple *stick-and-peel* procedure. (**b**) SEM image of a transferred convex Al NHA circular pattern on the flexible PSA tape.

The 500-nm-pitch Al NHA curved film covering the 2D microlens array on PC CD substrate was also successfully transferred onto a Scotch tape by a *stick-and-peel* procedure [12], illustrated schematically in Figure 6a. This produced convex (outward curved) nanopatterned Al film structures, as seen in the SEM photograph of Figure 6b. Good film uniformity over a large area is observed (surface grooves and other surface inhomogeneities are attributed to the original CD surface from which the Al film was detached). The flexibility of the Scotch tape support increases the versatility of the structure, adding further curving and adhesive functionalities to the already curvilinear nanopatterned Al film.

## 4. Discussion

It should be noted that the film/surface shaping technology demonstrated here is suitable to be applied to a variety of thin film materials. In addition to Al, other metals (e.g., Au, Ti, Cr, Ni) can be deposited onto PC CD substrates by well-known deposition methods as thin films that allow 50 keV incident electrons to penetrate into the PC material. Similarly, besides PC, other polymeric materials prone to crosslinking under electron irradiation and capable of hosting thin films (e.g., poly(methylmethacrylate) [14]) could be used as substrates. The use of PC CD is particularly remarkable because it is a highly available, ready-to-use, versatile and cost-effective material support.

For a given e-beam energy, the shaping limitations of the proposed method are determined by a variety of material and geometrical issues. Chemical composition and molecular structure of the substrate affect the penetration and absorbed energy of the incident e-beam and the maximum volume shrinkage (crosslinking) achievable in the irradiated polymer region. The film material composition and thickness also influence the penetration range and depth of the e-beam into the substrate. Even if the highest volume shrinkage is obtained, film-substrate adhesion strength, substrate elastic modulus and stiffness and pattern geometry of the deposited film impose a limit to the maximum surface/film curvature. Therefore, all these factors should be taken into consideration when selecting both, substrate and film materials for a particular application.

Finally, it should be mentioned that, although the conventional single-EBL direct-write system used in this work is not suitable for mass production due to its slow writing speed capacities, recent advances in the development of massively parallel electron beam direct-write (MPEBDW) systems [15] are expected to address this roadblock.

## 5. Conclusions

We have introduced a non-contact method to create non-planar nanopatterned thin films on PC CD substrates. Al NHA films on PC CD supports have been curved by direct e-beam irradiation of the film region to be shaped. Electron irradiation crosslinks the PC region under the Al film, shrinking it and deforming the film. The curvature shape and dimensions can be controlled by the irradiated layout dimensions and the electron dose. The technique has been used to fabricate a 2D microlens array on an Al NHA film, which exhibits both, plasmonic characteristics due to the metal NHA and optical features of diverging lenses. In addition, the concave optical elements of the array can be used as liquid micro-reservoirs. We have also demonstrated that the curved nanopatterned Al films can be easily transferred onto a conventional PSA tape. The non-contact property and design flexibility offered by e-beam direct writing, the film transfer capability onto soft malleable supports and the possibility of being applied to a variety of thin film and substrate materials make the presented shaping technology highly promising for implementing multifunctional micro/nano-devices and systems on both rigid (e.g., PC CD) and flexible (e.g., Scotch tape) polymeric substrates.
